# Supplementary figures and images for: An acute dose of intranasal oxytocin rapidly increases maternal communication and maintains maternal care in primiparous postpartum California mice
Source: PLoS One. 2021 Apr 22;16(4):e0244033. doi: 10.1371/journal.pone.0244033 (PMC8061985; doi:10.1371/journal.pone.0244033)

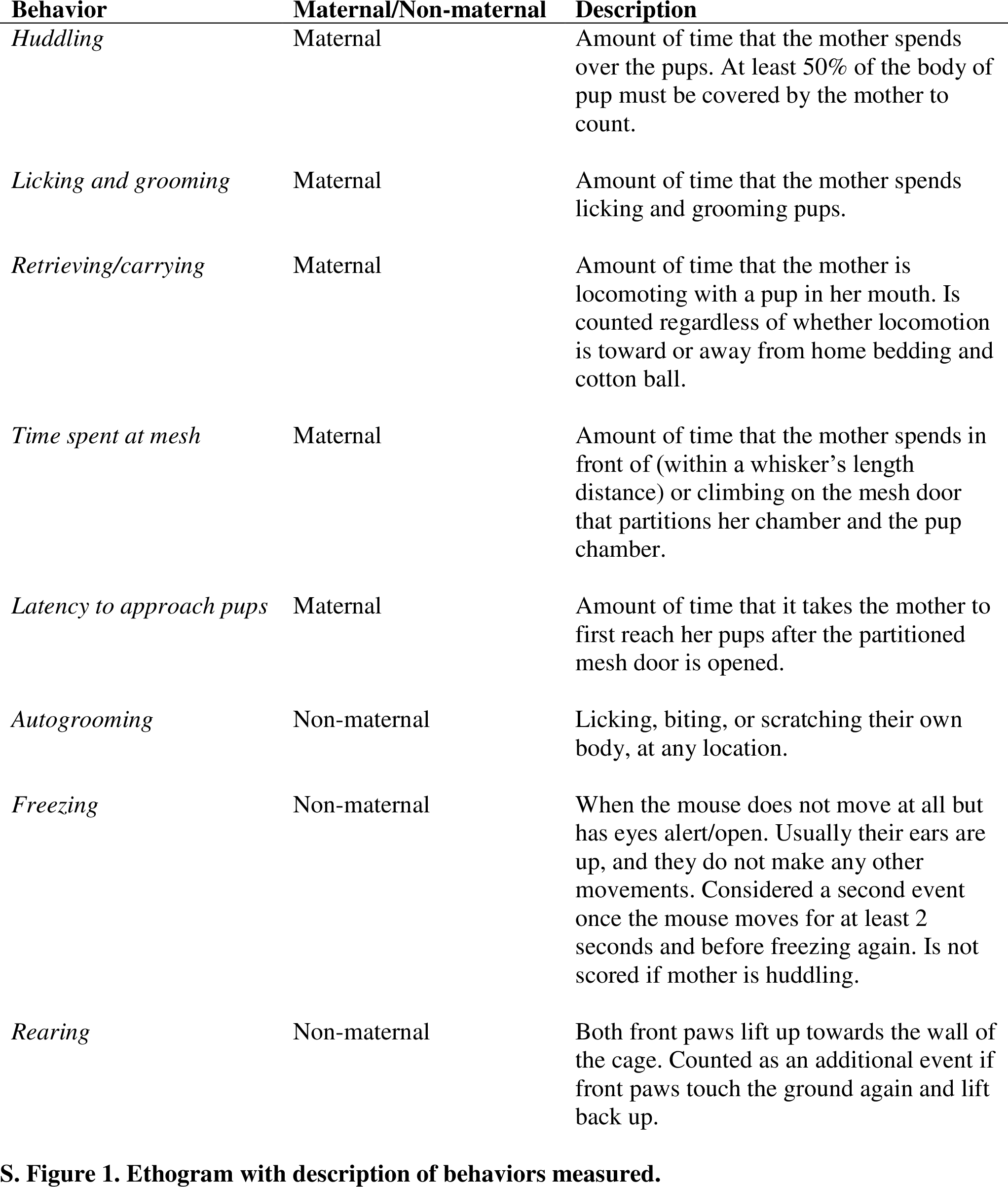

Supplement: S1 Fig — (TIF) [file pone.0244033.s001.tif]

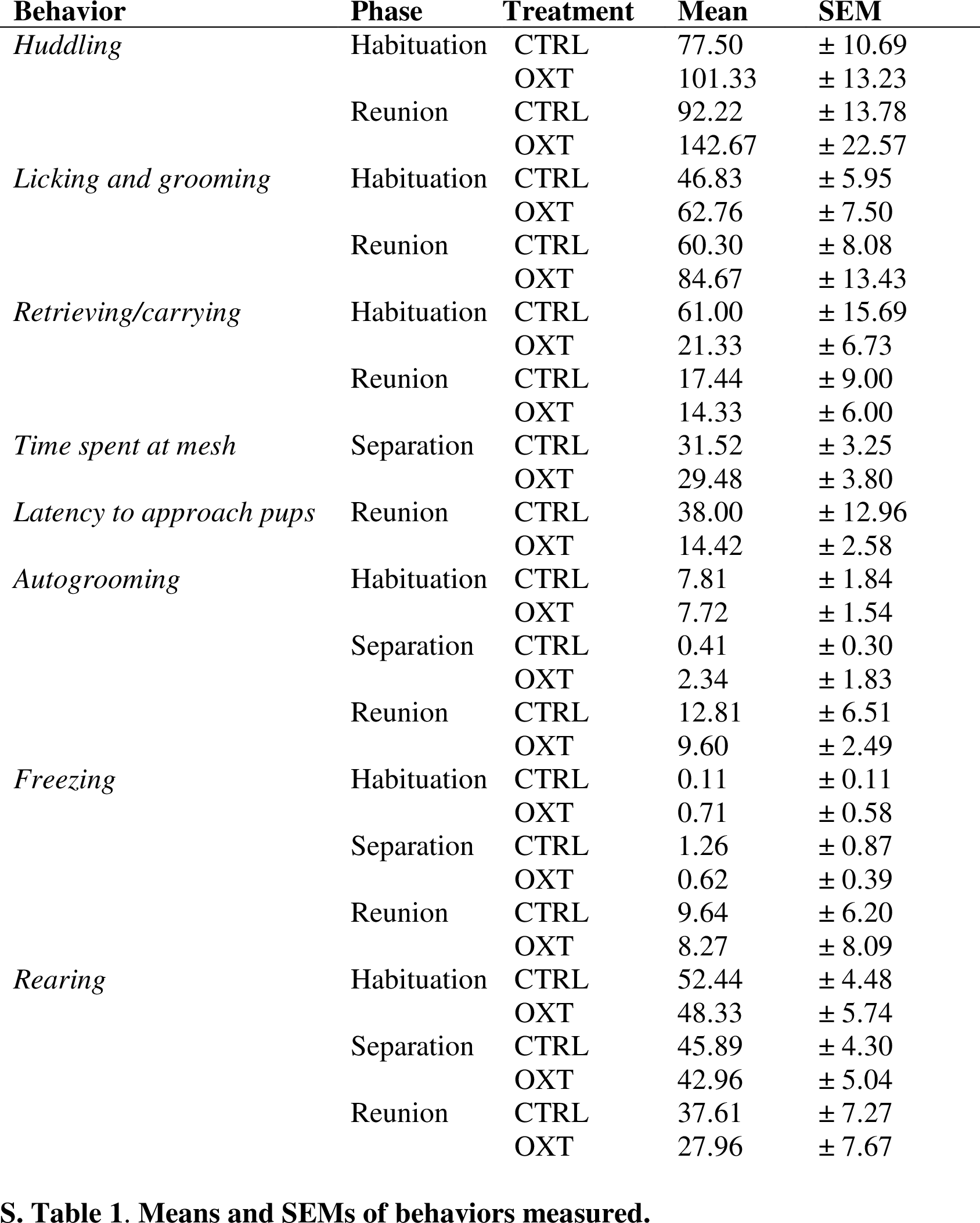

Supplement: S1 Table — (TIF) [file pone.0244033.s002.tif]
