# Supplementary material for: An acute dose of intranasal oxytocin rapidly increases maternal communication and maintains maternal care in primiparous postpartum California mice
Source: PLoS One. 2021 Apr 22;16(4):e0244033. doi: 10.1371/journal.pone.0244033 (PMC8061985; doi:10.1371/journal.pone.0244033)
Supplement: Attachment — Submitted filename: PLOS ONE Review Comments Rd2.docx [file pone.0244033.s004.docx]

Please use the space provided to explain your answers to the questions above. You may also include additional comments for the author, including concerns about dual publication, research ethics, or publication ethics. (Please upload your review as an attachment if it exceeds 20,000 characters)

Reviewer #1: All of my comments have been addressed in the revisions. The authors did a very nice job, this is a great contribution.

Reviewer #2: (No Response)

Reviewer #3: The authors were responsive to my previous comments, and the manuscript has improved. However, I have quite a few additional suggestions for increasing the clarify of the writing.

L12 – Mention saline controls.

**L12—Amended**

L19 - Not clear what “Maternal-pup USVs were correlated upon reunion” means – the number of USVs? Frequency? Timing? (Also, “Maternal-pup USVs” itself isn’t clear; try using something like “Mothers’ and pups’ USVs” instead.)

**L19—Amended**

L20 – “more positive change” is confusing. Change to something like “larger increase”?

**L21—Amended**

L24 – Change “single dose of” to “single treatment with”?

**L28—Amended**

L32 – Explain “pup whines.”

**L38—Amended**

L46-48 – It’s somewhat misleading to say that OXT “likely plays an important role in… producing behaviors that support maternal care,” since we already know this statement to be true (as summarized in the following sentences.”

**L53—Removed likely**

L56-58 – The point about “many species” doesn’t follow from the previous sentences, which mention only rats and mice.

**L64—Amended**

L63 – As I mentioned in my previous review, I find the phrase “both non-lactating and lactating maternally-experienced females” confusing because it can be interpreted to mean that only the lactating females were maternally experienced. Maybe try rephrasing as “maternally experienced females that were either lactating or non-lactating”.

**L69-70—Amended**

L68-69 – This sentence is very similar to the one at the end of the previous paragraph, which raises the question of why these two paragraphs are separate. Also, the first sentence of this paragraph (L59-60) seems to imply that the paragraph will focus on OXT receptors, which isn’t true of the second half of the paragraph. Consider removing the two sentences on receptors, since you didn’t quantify receptors in this study, and either reorganizing or consolidating the remainder of this paragraph and the previous paragraph.

**L64-74—Amended**

L84 – Consider starting a new paragraph with the sentence beginning “A key social behavior…” Also, you might want to add something like “in the context of OXT” after “not been measured”.

**L104—Amended**

L98 – Pultorak et al. 2017 is cited twice.

**L117—Amended**

L104 – Change “have” to either “can have” or “has.”

**L123—Amended**

L120 – Maybe change “responses” to “responsiveness” to make the statement a little broader? As is, it seems to refer only to the CRH and DEX challenges mentioned earlier in the same sentence.

**L104—Amended**

L120-121 – The description of pair housing/family composition is out of order, since you don’t mention the actual pairing of animals until after the next sentence.

**L140—Amended**

L124 – Again, it’s a little confusing to mention that pairs produced offspring before talking about when females were visibly pregnant (subsequent sentence).

**L144-145—Amended**

L150 – Has IN treatment been shown to be less stressful than ICV treatment? If not, please indicate that this is presumed to be the case, rather than stating it as a fact.

**L172—Amended**

L224 – The first part of this sentence is confusing. Do you mean that the videos were scored in random order (rather than the behaviors themselves)?

**L246—Amended**

L294-297 – This seems backward. If scores from the reunion phase were subtracted from scores from the habituation phase, then positive difference scores indicate that habituation-phase scores were higher than reunion-phase scores.

**L318—Amended**

L340-341 – The point about “both consistency and differences” is confusing, and the sentence overall isn’t particularly informative. I suggest that you delete it.

**L364-365—Amended**

L347 – It’s not essential, but I think it would be helpful to specify that you’re referring to the number of maternal and pup calls (rather than some other measure). The same point applies to several other parts of the manuscript, including L408-411 and L440-441.

**L371, 436—Amended**

L396-403 – The first sentence of this paragraph indicates that the following analyses focus on effects of IN OXT, but the next two sentences sound like they’re describing within-subjects changes (or lack thereof) over time rather than effects of IN OXT. Please clarify.

**L426—Amended**

L474 – As described in the methods, mothers had “multisensory pup experience,” although without touch, during the separation phase. Therefore, it would be more appropriate to emphasize the presence or absence of tactile stimuli in this sentence, rather than including this point only parenthetically. Same point for L480.

**L502. 508—Amended**

L477 – Delete “in” and “be.”

**L505—Amended**

L561-563 – Which species do these references refer to?

**L595-596—Amended**
